# Investigation of the Bending Process and Theory in Free-Boundary Pneumatic Film-Forming for Curved Image Sensors

**DOI:** 10.3390/s24196428

**Published:** 2024-10-04

**Authors:** Weihan Zheng, Chunlai Li, Jiangcheng Hu, Liang Guo

**Affiliations:** 1 Changchun Institute of Optics, Fine Mechanics and Physics, Chinese Academy of Sciences, Changchun 130031, China; 19158767130@163.com (W.Z.); 15040873714@163.com (C.L.); hujiangcheng22@mails.ucas.ac.cn (J.H.); 2 University of Chinese Academy of Sciences, Beijing 100049, China

**Keywords:** curved image sensors, pneumatic film, free boundary, bending process, bending theory

## Abstract

To explore the bending process and theory of the free-boundary aerodynamic film forming method for curved detectors, this study integrates practical forming structures with theoretical analysis and establishes a simulation model to investigate stress, strain, and morphological changes during bending. The analysis indicates that the shift from “projection” to “wrapping” in forming theory is due to the release of boundary degrees of freedom. The forming process can be summarized as the mold’s arc characteristics, originating from the chip’s corners, gradually replacing the chip’s rectangular characteristics along the diagonal, resulting in corresponding stress and strain changes. The “wrapping” bending theory of this method has significant advantages over traditional methods and represents a crucial direction for achieving higher curvature in the future. However, this study found that the use of film pressure can only inhibit out-of-plane deformation to a certain extent, and the buckling phenomenon will still occur when the thinner chip is bent. It prevents the use of thinner chips in the thinning–bending method, so avoiding out-of-plane deformation during the molding process is the direction that needs to be broken in the future.

## 1. Introduction

**Figure 1:**
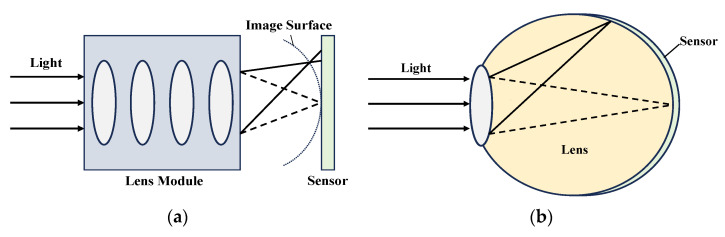
Principle comparison of imaging methods: (**a**) distortion principle of traditional image sensor due to Petzval field curve; (**b**) imaging principle of curved image sensor inspired by human eye.

Currently, commercial camera image sensors are primarily based on planar thin-film or wafer technologies. However, after light passes through the lens module, Petzval field curvature can cause off-axis light to image on a curved surface, resulting in distortion at the corners [1,2], as shown in Figure 1a. To ensure image quality, it is necessary to incorporate numerous lenses and mirrors into the optical system to correct for field curvature and minimize aberrations.

For example, the Schmidt–Cassegrain design of the Hubble Space Telescope and the Kepler Space Telescope uses numerous mirrors to correct aberrations [3,4]. A camera with a 120° wide field of view typically requires eight to 13 lenses in its optical system to meet imaging standards [5]. This accumulation of lenses conflicts with the volume and weight constraints of aerospace equipment, limiting the use of larger, brighter, and higher-performance lens modules.

In smartphones, as the semiconductor technology advances and chip integration has been increasing, the overall thickness of phones has been decreasing, yet the thickness of the lens section remains stubbornly high. For instance, the thickness of the iPhone has risen from 6.9 mm in the sixth generation to 8.25 mm in the 15th generation. The impact of compensatory lenses on lens thickness cannot be overlooked.

Inspired by the structure of the human eye, the researchers found that using curved image sensors in cameras was the most straightforward way to solve the problem [6,7], as shown in Figure 1b. This has led to the development of curved sensors with wide fields of view, deep depth of field, and aberration-free imaging capabilities [8]. Additionally, the reduction in the number of lenses has improved color correction.

Currently, there are three main methods for manufacturing curved image sensors: thinning–curving based on planar sensors, special-structure curving [9,10,11,12,13], and in situ growth based on curved surfaces [14,15]. Among these, the thinning–curving method for planar image sensors offers unmatched advantages in terms of resolution and industrialization potential compared to the other methods.

Dumas et al. utilized a 50 μm thick silicon-based device to create a detector array with a curvature radius of 80 mm, achieving this without significant degradation in photoelectric performance. This study demonstrates that thinning and curving existing planar sensors to form curved detectors is indeed feasible [16]. Gregory et al. applied their double-punch thinning process to backlit CCDs, reducing the total device thickness to 200 μm and achieving a detector curvature of 5.44 mm, all while ensuring a yield of over 95% [17]. B. Chambion et al. fixed the sensor chip onto a semicircular plate and controlled the plate’s movement with air pressure, allowing a 100 μm thick CMOS sensor to achieve curvature radii ranging from +∞ to 280 mm [18]. Guenter et al. utilized the higher compressive strength of single-crystal silicon over tensile strength, altering the bending theory to produce a chip curvature primarily from the compressive strain. They achieved a curvature radius of 16.7 mm using a free-boundary pneumatic film molding method, which is twice that of other methods, and has superior surface accuracy [19].

However, Guenter and colleagues did not provide detailed descriptions of the bending theory during molding or variations in stress and strain, which are important for future method optimization. This study simulates the molding process of sensor chips to analyze changes in shape, stress, and strain, exploring the molding theory and identifying existing issues.

## 2. Bending Theory

**Figure 2:**
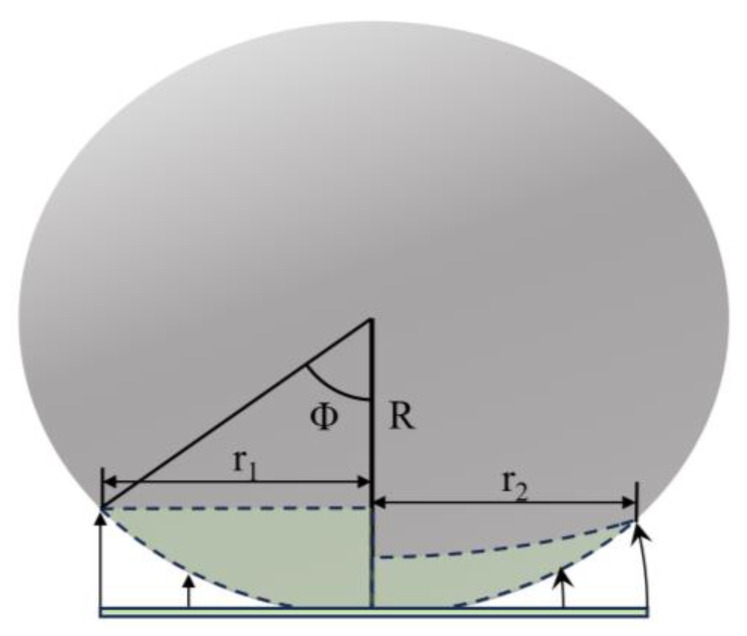
Forming principle; the left side is the traditional fixed-boundary mold forming, while the right side is the free-boundary pneumatic film forming.

The free-boundary pneumatic film forming technique significantly contrasts with the traditional fixed-boundary mold extrusion process, involving two different forming theories, as shown in Figure 2. On the left side of the figure, the traditional method is shown, where the chip is projected onto a spherical surface, maintaining a constant radial distance r1. Curvature is mainly achieved through radial strain, with an average radial strain $\varepsilon_{\varphi }=\left[\varphi -\sin\left(\varphi \right)\right]/\varphi$ (all radial and circumferential strains in this paper refer to chips) [17]. In contrast, the right side depicts the model described in this paper, which fits to the spherical surface in a wrapped form. The average circumferential strain $\varepsilon_{\varphi }=-\left[\varphi -\sin\left(\varphi \right)\right]/\varphi$ is always negative, mainly creating curvature through a circumferential compressive strain [17]. Comparing the polar diameter and average strain formulas for both methods, it is evident that the proposed method produces smaller strains for the same curvature, allowing for greater curvature.

**Figure 3:**
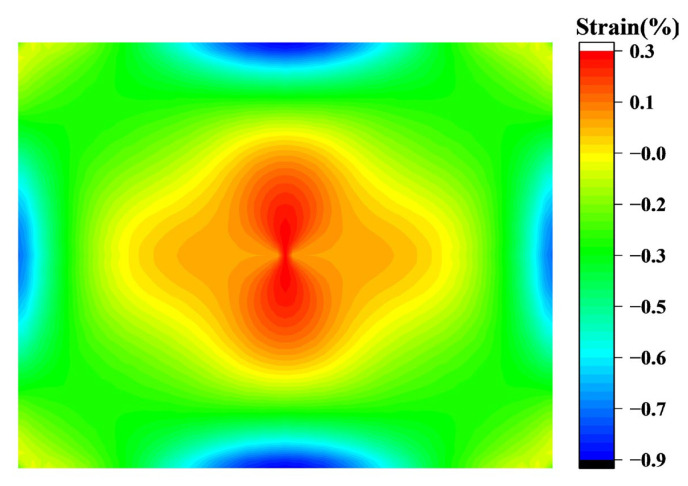
When using free-boundary mold molding, the molding method has been changed from “projection” to “wrap”.

**Figure 4:**
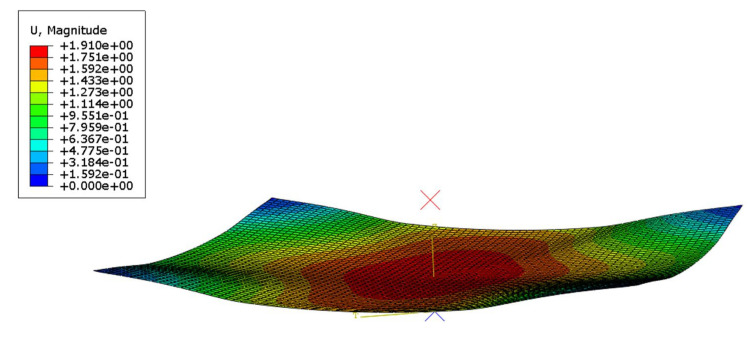
If the difference between the displacement in the x and y direction and the displacement in diagonal direction is too large, there will be a tendency of out-of-plane deformation.

The transition from the traditional projection-based method to the wrapping method is attributed to the release of edge degrees of freedom. However, using free-boundary mold forming, only releasing chip DOF (degree of freedom) without making improvements, will create other problems; although chip curvature is also mainly generated by circumferential compressive strain, as shown in Figure 3, this method will increase molding instability when thinner chips are used, making it prone to out-of-plane deformation, as depicted in Figure 4.

**Figure 5:**
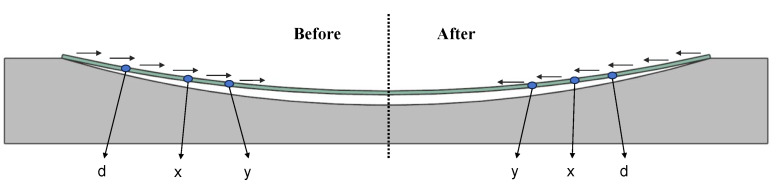
Points x, y and d in the figure represent the intersection of the dividing line and the lines in the x, y and d directions. Make the x, y, and d points on the fitting boundary located on the same section diagram. The points representing the diagonal direction on the fitting boundary gradually approach and exceed the x and y points during the forming process. It indicates that the fitting speed in the d direction is faster than that in the x and y directions.

This is contrary to the pursuit of lower chip thickness, which is the key to achieving higher curvature in the thinning–bending method (the thinner the chip, the less bending stress, the less damage, and the easier to achieve higher curvature). Therefore, Guenter and his colleagues used a thin film to apply pressure so that the entire chip surface is stressed simultaneously. To some extent, this method mitigates the tendency for diagonal buckling that occurs when the edges contact the mold, introducing a diagonal fit direction that reflects the mold’s circular characteristics. As shown in Figure 5, fitting in this direction is faster than fitting in the x and y directions, which results in the chip’s rectangular features being gradually replaced (the rectangular characteristic refers to the long and the short sides of the chip as the leading bending factors, and the curvature is formed from the edge in a form similar to that of pressing the beam into the mold, so that the fitting boundary line between the chip and the mold remains rectangular from the plane perspective). The rectangular characteristic refers to the long and short sides of the chip as the primary bending factors, with the curvature forming from the edge in a manner similar to pressing a beam into the mold. As a result, the fitting boundary line between the chip and the mold remains rectangular from a planar perspective.

## 3. Setup of Simulation Parameters

**Figure 6:**
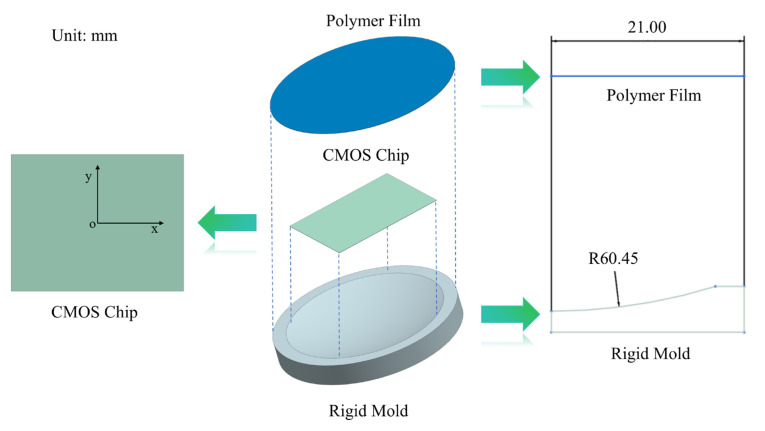
Structure diagram of free-boundary pneumatic film forming method.

The simulation model consists of three components: a polymer film, a CMOS chip, and a rigid mold, as shown in Figure 6. During processing, the chip is placed horizontally in the rigid mold, supported at four points without constraints on the edges. The polymer film is positioned above the chip, with one side in contact with the chip and the other connected to an air pump. As air pressure gradually increases, the film causes the chip to bend. The following section will discuss parameter selection for these three components.

### 3.1. Elastic Modulus for Polymer Films

First, the parameter selection for the polymer film is discussed. As the component that directly applies pressure to the chip, its elastic modulus is the most critical influencing factor. This study examined the effects of polymer films with different elastic moduli on the normal forming of the chip, and the results showed that variations in elastic modulus have a minimal impact on the forming stress of the chip, as shown in Table 1. Therefore, a common polyethylene material with an elastic modulus of 400 MPa was chosen for the film.

### 3.2. Parameters of CMOS Chips

In the study by Guenter et al., it was found that simulating the CMOS chip as a single crystal silicon chip yielded results consistent with experimental data. Therefore, for computational convenience, the CMOS chip is simplified to a single-crystal silicon chip with dimensions of 28 mm × 22.3 mm and a thickness of 0.06 mm. Single-crystal silicon demonstrates significant anisotropy. Studies indicate that ignoring this anisotropy and using isotropic parameters can introduce errors of approximately 10%. By using the average Poisson’s ratio and Young’s modulus values, the error in calculating the central deflection can be reduced to around 1% [20]. To simplify the model while maintaining accuracy, the average Young’s modulus E_a_ and average Poisson’s ratio μ_a_ are calculated using the primary crystallographic direction of the commonly processed (1, 0, 0) plane of single-crystal silicon. The results are E_a_ = 156 GPa and μ_a_ = 0.23 [21,22].

### 3.3. Selection of Mould Materials

The requirements for rigid mold materials include three key factors: a sufficiently high elastic modulus to ensure that no deformation occurs during the pressing of the chip; a low thermal expansion coefficient to prevent distortion during the high-temperature curing of the epoxy resin; and a sufficiently smooth surface. These characteristics align with the demands of traditional forming methods, allowing for the selection of materials such as glass and ceramics. The inner surface curvature of the mold is 60.45 mm, and the friction coefficient is set at 0.005.

## 4. Process of Bending

The bending process consists of two phases: the Edge fitting Stage and the Molding Stage. To facilitate the explanation, an XY coordinate system is established on the CMOS chip, as illustrated in Figure 6. Since the displacement of the chip’s center point O persists throughout the bending process, the displacement d of point O is used as the measure of the entire forming progress. This point accumulates a displacement of 2.71 mm in the Z-direction throughout the entire process (subsequent displacements will also refer to the Z-direction).

### 4.1. Edge Fitting Stage

**Figure 7:**
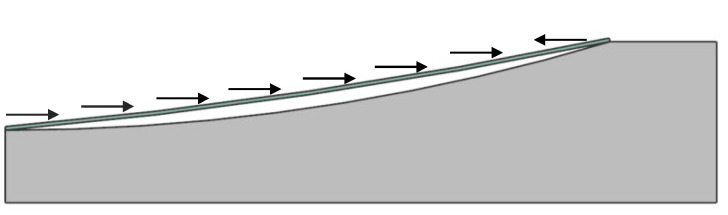
Edge fitting direction.

In this stage, the chip initially contacts the mold at the center of the long and short sides under the pressure of the film, and then gradually fits from the center to the corners as shown in Figure 7. The use of the film allows for greater displacement near the diagonal compared to using a mold, reducing the likelihood of diagonal buckling. This stage can be divided into two parts: central contact and progressive fitting. The use of the film allows for greater displacement near the diagonal compared to using a mold, thereby reducing the likelihood of diagonal buckling.

#### 4.1.1. Edge–Middle Contact

**Figure 8:**
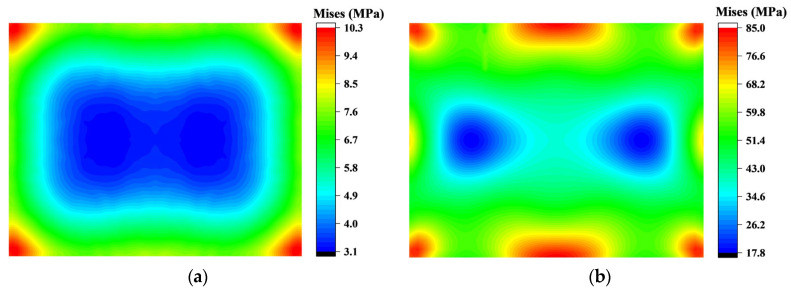
At the beginning of bending, the Mises stress was mainly affected by the length of the chip, and the Mises stress on the *y* axis increased to distinguish the low stress in the middle of the chip. (**a**) Before the division of the middle low-stress region; (**b**) After the segmentation of the low stress region in the middle.

Initially, during pressure application, the stress at the chip’s four corners, acting as support points for the thin film and mold, is the highest. As the bending process continues and the chip undergoes significant deformation, edge stress increases rapidly. When the long edge contacts the mold, the midpoint displacement of the long edge reaches 1.68 mm with a stress of 556 MPa, which is far greater than the stress at the corners. At this stage, the maximum displacement at the midpoint of the short edge is 0.97 mm, only half that of the long edge. Thus, during the long edge contact, the chip’s bending around the y-axis is much greater than around the *x*-axis, leading to a rapid increase in stress near the y-axis and the formation of a low-stress region, as shown in Figure 8. In-plane stress is primarily influenced by the long dimension.

After the long edge contact, the short edge’s midpoint continues to displace by 0.108 mm and makes contact with the mold. This displacement is approximately 2.6 times that of the center point O. Consequently, the low-stress region exhibits an opposite stress variation compared to the long edge contact process, with the separated region shifting towards the y-axis, causing the stress to decrease from 20 MPa to 9 MPa. The maximum stress remains concentrated at the midpoint of the long edge and gradually increases throughout the bending process. At this point, the displacement at point O has reached 1.74 mm, completing 64% of the entire molding process.

Throughout the contact process, both radial and circumferential compressive stresses are much greater than tensile stresses. The maximum circumferential compressive stress and strain consistently occur at the midpoint of the long edge, where stress increases most rapidly and reaches its highest value. This makes the midpoint of the edge during this stage the most vulnerable to failure in the entire molding process. Excessive thickness-to-length ratios can cause buckling at the edge midpoint. By this stage, from the point of displacement, this stage has completed 64% of the entire molding process; if the buckling is serious, it will lead to product scrap under rapid deformation. Only slight buckling with a small probability can be restored to the normal state by means of mold support after fitting with the mold. So, Guent et al. solved this problem by using a special mold [19].

#### 4.1.2. Edge Fitting

**Figure 9:**
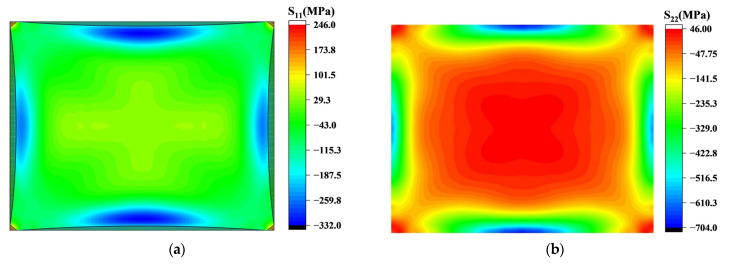
Diagram of stress distribution when edge fitting is completed (S11 represents radial stress, and S22 represents circumferential stress): (**a**) the shaded area indicates where bonding is complete, forming an arcuate boundary line near the edges. Stress concentration occurs in the central part of the unbonded arc; (**b**) circumferential stress distribution after edge fitting.

After the long and short sides of the chip contact the mold, they are further fitted from the contact point to the inner plane and both ends of the edge simultaneously, resulting in an arc distribution of the fitting boundary, as shown in Figure 9a. This also results in areas of stress concentration within the plane near the boundary. The stress in this region increases gradually with the edge fitting, and it is the region with the largest radial stress in the chip. The tension stress zone located in the center of the chip is affected by the chip size and stress concentration area, and is distributed along the *x* and *y* axes accordingly. During the edge fitting process, the circumferential stress gradually develops a distribution trend of compressive stress along the edge and tensile stress at the center of the chip, as shown in Figure 9b.

**Figure 10:**
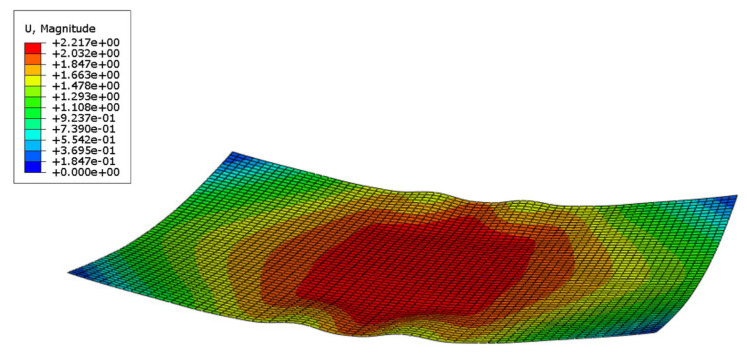
Buckling from the diagonal caused by the chip being too thin.

It is worth noting that when the chip thickness is reduced to 0.02 mm and the curvature radius is less than 70 mm, the buckling phenomenon not mentioned by Guenter et al. will occur at this stage, as shown in Figure 10. This buckling is similar to that produced by free-boundary mold forming in Section 2. This shows that the use of film compression can only alleviate the buckling from the four corners, but not completely solve the problem.

**Figure 11:**
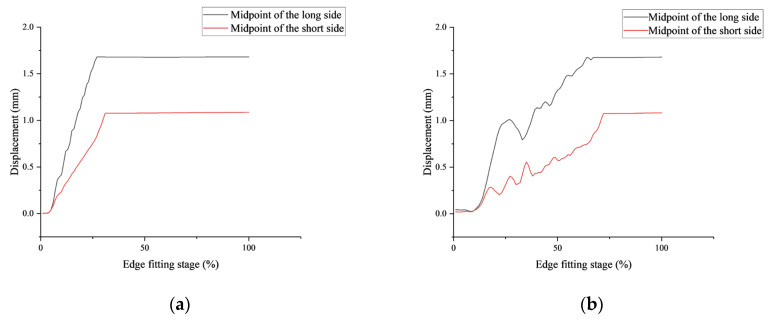
Repeated changes of the chip edge midpoint displacement and subsequent laminating progress reduce the edge fitting speed. (**a**) Displacement changes during normal forming process; (**b**) The change of displacement at the midpoint of the edge before buckling occurs.

Through analysis, it is found that this phenomenon arises due to the reduced thickness of the chip, which leads to an instability in the molding process. During the contact and bonding process, the chip edges undergo repeated motion (illustrated in Figure 11), which slows down the edge-fitting speed. Concurrently, the displacement at center point O increases steadily after the edges make contact, resulting in the shape shown in Figure 4. This shape undergoes compression from the midpoint directions of the long and short edges in subsequent molding (Figure 7), ultimately producing the formation shown in Figure 10. To resolve this issue, it is essential to enhance the edge-bonding speed and reduce the displacement speed at the chip center.

### 4.2. Molding Stage

**Figure 12:**
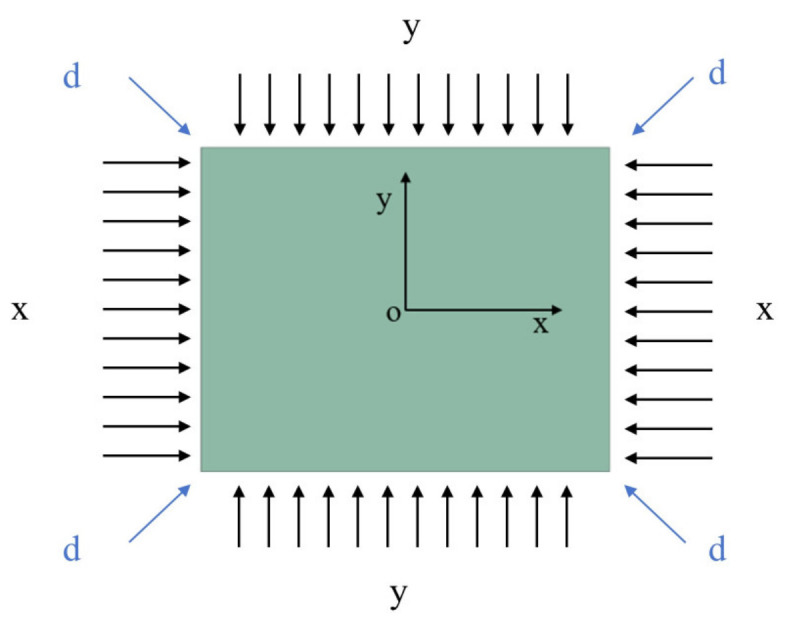
Three forming directions in the forming stage.

**Figure 13:**
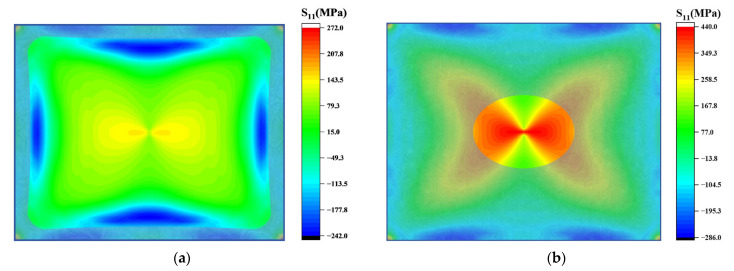
Affected by the direction of the diagonal fit, the shape of the fit boundary changes: (**a**) after the end of edge fitting for a period of time, the arc boundary line from the four corners gradually replaces the rectangular boundary line; (**b**) boundary shape near the end of molding.

To facilitate analysis, the fitting of the chip and mold at this stage is divided into three directions, x, y and d, as shown in Figure 12. As can be seen from Figure 5, the point representing d direction fitting on the boundary (hereinafter referred to as point d) rapidly approaches the point of x and y direction-fitting on the boundary (hereinafter referred to as point x and y) during the molding process. This indicates that the speed of d direction-fitting is much higher than that of x and y direction in this process. Moreover, since the d direction fitting originates from the four corners, the fitting boundary is an arc with the same curvature as the mold, which changes the shape of the boundary, as shown in Figure 13a. The subsequent molding will continue this process, with point d successively covering points x and y. In other words, the arc boundary at the four corners will progressively envelop the boundary from both the short side and the long side, ultimately completing the chip bending in the form of the arc boundary, as shown in Figure 13b. This can also be seen as the process of replacing the rectangular characteristics of the chip with the circular arc characteristics of the mold.

**Figure 14:**
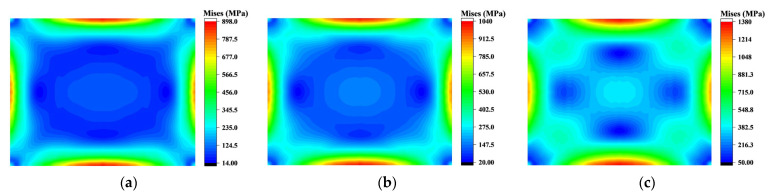
Mises stress distribution during molding process: (**a**) Mises stress distribution at the beginning of the molding process; (**b**) Mises stress distribution during molding; (**c**) Mises stress distribution change during molding process.

The change in equivalent stress during the molding process is relatively straightforward. Point O, the intersection of the two diagonals, experiences a more rapid increase in stress compared to other positions outside the edge, resulting in the central low-stress region becoming “segmented” into a ring shape, as shown in Figure 14a. Subsequently, this ring-shaped distribution is further segmented by stress near the diagonals, which grows slower than at the center but faster than in other parts of the low-stress region (Figure 14b). After several rounds of “segmentation,” the equivalent stress distribution is as shown in Figure 14c. The stress in the middle of the edge grows more significantly, and substantial stress has already accumulated during the edge fitting process. Therefore, the stress in the middle of the long edge remains the maximum until the end of molding.

**Figure 15:**
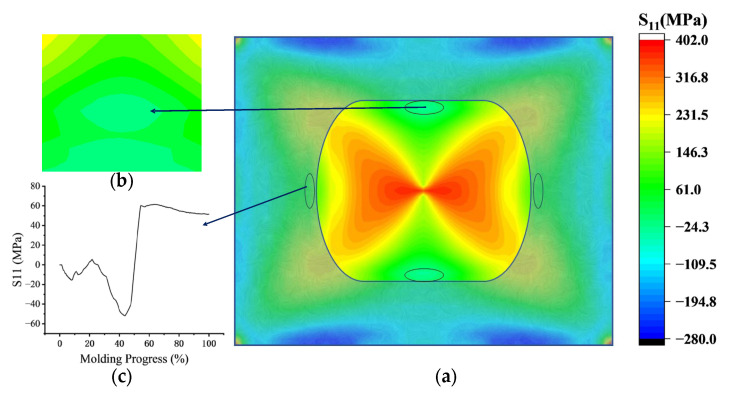
On the boundary point d passes point x and does not reach point y: (**a**) The circular boundary line from the four corners has just swallowed up the short boundary line diagram; (**b**) at this time, the compressive stress in this region reaches its peak, and the maximum stress and range in this region are smaller than that in the concentrated region near the edge; (**c**) at this time, the area indicated in Figure (**c**) has been fitted and there is no longer any stress concentration. Taking the region c in the Figure as an example, it is shown that the stress will produce a concentrated phenomenon of regional stress change process.

From the perspective of radial stress, after edge fitting, the region of maximum compressive stress (i.e., the stress concentration area) moves inward along the boundary line, with its extent and size gradually decreasing, as shown in Figure 9a, Figure 13, and Figure 15b. This occurs because the fitting speed in the diagonal direction is faster than in the x and y directions. The arc boundary at the corners completes the arc boundaries of the long and short edges, which alleviates stress concentration, as illustrated in Figure 15a. In this process, areas along the x and y axes that experience stress concentration exhibit similar stress variation trends: the compressive stress increases rapidly after edge fitting, reaching a peak when adjacent to the fitting boundary line (Figure 15b). Subsequently, it decreases at an even faster rate once the fitting is completed, with the overall trend illustrated in Figure 15c. The closer the stress concentration area is to the chip edge, the more pronounced the phenomenon and the faster the rate of change in compressive stress.

**Figure 16:**
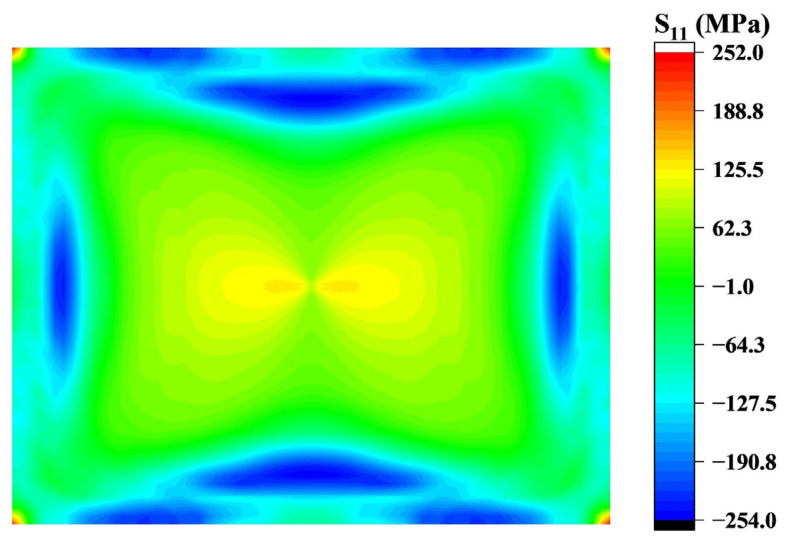
In the center of the chip appears a droplet area symmetrical about the *y*-axis.

**Figure 17:**
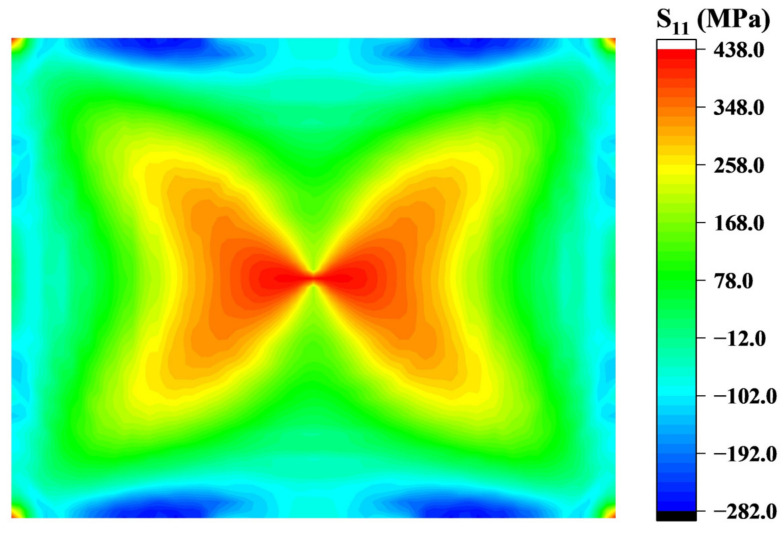
Radial stress distribution at the end of molding.

Radial tensile stress variation is primarily influenced by three factors: compressive stress in the stress concentration region, tensile stress generated at the corners as support points (where stress at the corners transitions from compressive to tensile after bonding with the mold), and the effect of chip size. At the end of edge fitting, the compressive stress in the stress concentration area is significantly higher than the stress at the corners, causing the central tensile stress region to distribute along the *x* and *y* axes as shown in Figure 9a. As the chip continues to fit, due to unequal edge lengths, a pair of water-drop regions symmetrical about the *y*-axis are generated in the middle of the chip as shown in Figure 16. Additionally, with the reduction in stress concentration effects, tensile stress at the corners increases rapidly. The droplet-shaped region and its surrounding tensile stress area start to extend towards the corners. Eventually, once the stress concentration area at the midpoint of the boundary line completely disappears, the tensile stress becomes fully dominated by the corners and is distributed along the diagonals, as shown in Figure 17.

The circumferential stress distribution of the chip when edge fitting is completed is shown in Figure 17. On this basis, as the boundary line advances, the tensile stress in the chip transitions from the outside to the inside, becoming compressive stress, while the compressive stress decreases. Thus, the center area of the chip remains under tensile stress. Through observation, it is found that the longitudinal displacement of O point reaches 2.69 mm when no compressive stress is generated after the chip is fitted, which completes 99% of the forming process compared with the total displacement of 2.71 mm. The strain distribution is similar to the stress, and the circumferential strain is much larger than the radial strain, so the chip curvature is mainly caused by the circumferential compressive strain.

**Figure 18:**
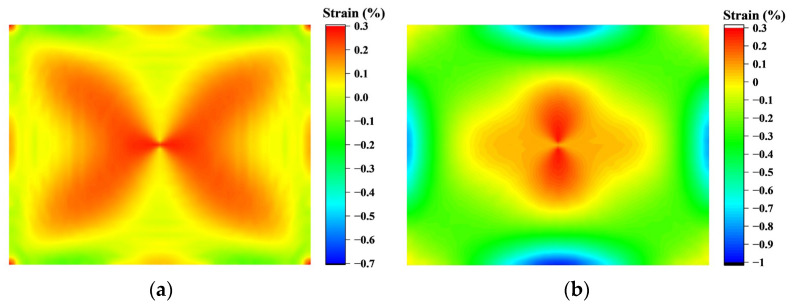
Strain distribution in the chip after molding: (**a**) radial strain results; (**b**) tangential strain results.

The final molding results are shown in Figure 18a,b. The strain cloud map is similar to the DIC results and the simulation results of the bending test conducted by Brian Guenter et al. using special molds [19]; only the strain in the middle of the edge is slightly larger than the result. However, this position is the maximum strain position, which does not affect the overall change law of stress and strain in the chip. Moreover, the 0.9% strain is significantly lower than the uniaxial 8.5% and biaxial 3% strains achieved by MG Masteghin et al. [23], as well as the 3% strain limit reached by Ming Chen et al. [24] at the micron scale, which does not affect the final curvature. The remaining positional strain is consistent with Brian Guenter et al. Therefore, the stress, strain, shape, change trend, and forming theory obtained based on this model are accurate enough.

## 5. Summarize the Outlook

In this study, the forming principle is integrated with the implementation structure, revealing that the transformation of the forming theory is attributed to the release of boundary freedom. The use of a thin film serves to restrain the out-of-plane deformation of the chip. Subsequently, the free-boundary pneumatic film forming method is simulated to explore the realization process of the free-boundary forming principle, as well as the stress, strain, and morphological changes occurring during the bending process of the planar image sensor. The process can be summarized as follows: “The chip and the mold are fitted from the outside to the inside, and the circular characteristics of the mold at the four corners replace the rectangular characteristics of the chip. Therefore, the radial stress concentration area gradually disappears, and the boundary line of circumferential pressure and tension strain changes from a rounded rectangle to an arc.” At the same time, it is found in the simulation that the use of thin film to pressurize the whole plane of the chip can only alleviate the out-of-plane deformation to a certain extent, and the chip will still produce buckling phenomenon when it is further thinned.

The free boundary pneumatic film forming method brings the realizable curvature to a new height from the bending theory, but new breakthroughs are needed to restrain the out-of-plane deformation of the chip. On the basis of the free boundary, future research can further explore the method of ensuring normal molding while using a chip with a smaller thickness to achieve higher curvature

## Figures and Tables

**Table 1:** The maximum equivalent stress within the chip for films with varying elastic modulus (MPa).

E	10	100	300	500	800	1200	3000
Mises	1375	1375	1373	1375	1377	1375	1376

## Data Availability

The data presented in this study are available on request from the corresponding author. The data are not publicly available due to privacy or ethical restrictions.
